# Applicability of the Chinese weight self-stigma questionnaire and its relationships with depression, physical exercise, and quality of life

**DOI:** 10.1038/s41598-025-07951-1

**Published:** 2025-07-02

**Authors:** Yuchao Fu, Jixiang Liu, Wei Lyu

**Affiliations:** 1 Changzhi University, Changzhi, Shanxi China; 2 Al-Farabi Kazakh National University, Almaty, Kazakhstan

**Keywords:** Weight self-stigma, Physical exercise, Depression-anxiety-stress, Quality of life, College students, Psychology, Human behaviour, Quality of life, Weight management

## Abstract

This study aims to validate the psychometric properties of the Chinese College Students’ Weight Self-Stigma Questionnaire (WSSQ), with a focus on examining its cross-cultural adaptability to provide evidence for localized measurement tools. It further seeks to explore the mechanism through which weight self-stigma influences quality of life satisfaction, elucidating the chain mediating effects of physical exercise (behavior) and depression-anxiety-stress (psychology) to fill the gap in mechanistic research on these pathways. Study 1 assessed validity and reliability via item analysis, exploratory factor analysis (EFA), and confirmatory factor analysis (CFA). Study 2 used the WSSQ (two dimensions: self-devaluation, Fear of Enacted Stigma), physical exercise scale (frequency, intensity, duration), DASS-21 (three subscales), and life satisfaction scale to construct structural equation models for testing chain mediation. Study 1’s EFA extracted two factors (self-devaluation, Fear of Enacted Stigma) explaining 63.05% variance. CFA showed excellent fit (χ^2^/df = 1.22, RMSEA = 0.032, CFI = 0.992). Convergent validity (AVE = 0.562/0.605; CR = 0.884/0.901) and discriminant validity (AVE square root > inter-factor correlations) met standards, with strong internal consistency (Cronbach’s α = 0.899). Study 2 found weight self-stigma negatively predicted life satisfaction (β = -0.390, *p* < 0.001), with significant chain mediation via physical exercise and depression-anxiety-stress (indirect effect = 0.059, 95% CI [0.011, 0.133]). The Chinese WSSQ demonstrated robust reliability and validity among college students. Weight management interventions should target weight self-stigma reduction, fostering “psychology-behaviour-health” virtuous cycles through physical exercise promotion and mental health improvement to enhance life satisfaction. The scale serves as a valuable tool for weight-related research.

## Introduction

Weight self-stigma denotes the internalisation of societal biases against obesity, whereby individuals adopt negative cognitions, evaluations, and feelings towards themselves, translating external discrimination into self-devaluation^1–3^. This process involves integrating societal weight-related negativity into self-perception, giving rise to emotions and behaviours characterised by self-criticism and self-rejection. Beyond compromising mental health, weight self-stigma may trigger cascading effects on behaviour and overall well-being^4^. Experiencing weight stigma often involves internalising stereotypes (e.g., perceiving overweight individuals as lazy), negative emotions (e.g., self-anger or dislike), and discriminatory attitudes, which are strongly linked to depression and anxiety^4–6^. For instance, studies in obese populations demonstrate that weight self-stigma correlates with elevated depressive symptoms, with obesity itself identified as a risk factor for poor mental health^7–9^. This association is partially explained by self-stigma’s erosion of self-esteem and self-efficacy, creating a cycle of psychological distress. The impact of weight self-stigma on behaviour is intertwined with body image and self-identity^10^. Individuals frequently fixate on perceived physical imperfections, leading to distorted body image perceptions—for example, obese individuals may erroneously believe they are universally viewed as unattractive, undermining social confidence and prompting withdrawal or unhealthy weight-control behaviours^11,12^.

As a factor with profound implications for individual physical and mental health, weight stigma has garnered significant attention in recent years. Various measurement tools have been developed to address this phenomenon, such as the Weight Bias Internalization Scale (WBIS) by Durso and Latner in 2008^14^, the Weight Self-Stigma Questionnaire (WSSQ) by Jason Lillis et al. in 2010^15^, and the Perceived Weight Stigmatization Scale (PWSS) by Atiqa Rafeh and Rubina Hanif et al. in 2019^16^. This study selected the WSSQ due to its strong reliability and validity. The WSSQ, developed by Jason Lillis in 2010, has been validated in countries such as Malaysia and Indonesia, demonstrating its reliability and effectiveness across different populations and cultural contexts. This makes it a widely recognized and trusted research tool, providing a solid empirical foundation for this study. The adaptation of this scale for Chinese college students is particularly important, as the number of overweight and obese individuals in China is rapidly increasing. According to the “Report on Nutrition and Chronic Disease Status of Chinese Residents (2020)”, the rates of overweight and obesity among Chinese adults aged 18 and above are 34.3% and 16.4%, respectively, with obesity rates on the rise, making obesity prevention and control an urgent priority^16^. To address this, the National Health Commission of China released the “Dietary Guidelines for Adult Obesity (2024 Edition)” on February 7, 2024^18^. The “Obesity Diagnosis and Treatment Guidelines (2024)” identify three key factors contributing to obesity^18^: (1) Changes in dietary structure: With rapid economic development, food supply has become more diverse, leading to increased consumption of high-calorie, high-fat, and high-sugar foods such as fast food, snacks, and beverages, while intake of fiber- and vitamin-rich foods like vegetables and fruits has decreased. Additionally, increased dining out has led to higher consumption of oily and salty foods, contributing to excessive energy intake. (2) Lifestyle changes: Economic growth has transformed transportation, with people increasingly relying on cars and subways, reducing opportunities for walking or cycling. Meanwhile, mechanization and automation have significantly decreased physical activity in the workplace, with many jobs involving prolonged sitting, leading to reduced energy expenditure. 3.Shifts in consumer attitudes: Increased disposable income has expanded leisure and entertainment options. However, activities such as watching TV and playing video games are sedentary, contributing to weight gain.

Against this backdrop, the study proposes the following hypotheses:

**Hypothesis 1:** The Chinese WSSQ exhibits robust reliability and validity among college students, including structural, convergent, discriminant, and criterion validity.

**Hypothesis 2:**Weight self-stigma is negatively associated with life satisfaction, such that higher self-stigma correlates with lower satisfaction.

**Hypothesis 3:**Physical exercise mediates the relationship between weight self-stigma and life satisfaction, with self-stigma influencing satisfaction via reduced exercise.

**Hypothesis 4:**Depression, anxiety, and stress mediate the relationship, whereby self-stigma lowers satisfaction through exacerbating negative emotions.

**Hypothesis 5:**A chain mediation pathway exists, where weight self-stigma reduces physical exercise, amplifies depression-anxiety-stress, and ultimately decreases life satisfaction.

By validating the reliability and validity of the WSSQ, this study aims to provide researchers and healthcare professionals with an empirically tested tool for assessing weight self-stigma among Chinese college students. This will not only help reveal the true psychological state and needs of college students but also explore the relationships between weight self-stigma, depression, physical exercise, and life satisfaction, thereby reducing the risks of depression and suicide. In future work, this scale will enable more precise mental health services and interventions for Chinese college students, effectively helping them address and resolve psychological issues in a timely manner.

## Participants and methods

### Participants

Sample Size Estimation: For exploratory factor analysis (EFA), statistical standards recommend a sample size of at least 5 times the number of items or a minimum of 150 participants^19,20^. For confirmatory factor analysis (CFA), the sample size should be at least 10 times the number of items^20^. The WSSQ consists of 12 items, so the ideal sample size for EFA should be above 150, and for CFA, it should be above 120.

In Study 1, a single sampling survey was conducted, and the obtained sample was randomly divided into two parts: Sample 1 was used for item analysis and EFA, while Sample 2 was used for CFA and cross-validation of the scale structure derived from EFA. A total of 460 college students were recruited for the WSSQ measurement. After screening the collected questionnaires based on response time or identical responses, 21 invalid questionnaires were excluded, resulting in 439 valid questionnaires and an effective response rate of 95.4%. As shown in Table 1, the sample included 211 males (48.06%) and 228 females (51.94%); 321 participants (73.12%) were aged 18–20, and 118 (26.88%) were aged 21–23; 261 participants (59.45%) were from science majors, and 178 (40.55%) were from humanities majors; 314 participants (71.53%) were from rural areas, and 125 (28.47%) were from urban areas.

In Study 2, four scales were used, with the maximum number of items in a single scale being 21. A total of 350 participants were included, meeting the minimum sample size requirement. After screening the collected questionnaires based on response time or identical responses, 12 invalid questionnaires were excluded, resulting in 338 valid questionnaires and an effective response rate of 96.6%. As shown in Table 1, the sample included 146 males (43.29%) and 192 females (56.80%); 271 participants (80.18%) were aged 18–20, and 61 (19.82%) were aged 21–23; 211 participants (62.43%) were from science majors, and 127 (37.57%) were from humanities majors; 264 participants (78.11%) were from rural areas, and 74 (21.89%) were from urban areas.

This study has been formally approved by the Ethics Committee of Changzhi University, with the number: CZU - IRB − 2024–001. All research methods follow the relevant guidelines and standards in the field of psychology. Cluster sampling is a sampling method that divides the population into several non - overlapping clusters, and then randomly selects some clusters as sample clusters, and investigates all individuals in the selected clusters. The reasons for choosing cluster sampling are as follows: first, it is relatively simple in actual operation, which can effectively reduce the complexity and cost in the sampling process. Especially in a research scenario like a school with obvious group divisions, it is convenient for the research team to coordinate and implement the survey with the school. Secondly, when the differences between individuals within the cluster are relatively large, cluster sampling can also ensure the diversity of the sample to a certain extent, thus improving the representativeness of the sample for the population. Before cluster sampling, we obtained the summary of physical examination data of each class through the school. These data only contain the average BMI of the class and do not involve individual privacy. We preferentially selected classes with an average BMI ≥ 24 as the sampling frame. This is to ensure that the proportion of obese individuals in the selected clusters (classes) is higher than the school - wide average level, so as to more targeted study the impact of obesity - related psychological factors on life satisfaction and improve the efficiency and effect of the research. On this basis, we selected a certain number of classes as research objects according to the principle of randomness. To ensure the authenticity and reliability of the survey results, we communicated with the school management before the start of the survey and obtained their formal permission to collect samples. To ensure the authenticity and reliability of the findings, we communicated with the school administrators before the commencement of the survey and received their official permission to collect the sample. Before filling out the questionnaire, the researcher explained the purpose, process and significance of the study in detail to the participants and answered all the queries raised by them, and made it clear that they had the right to voluntarily participate and withdraw from the study, and that they would not be adversely affected in any way as a result of their participation. Informed consent was obtained from all university student participants involved in this study, and the researchers supervised their independent completion of the questionnaire in a closed and quiet environment.

**Table 1:** Demographic characteristics. Source(s): author’s own creation

	Variable	Category	Number of Participants	Percentage
Study 1	Gender	Male	211	48.06%
		Female	228	51.94%
	Age	18–20	321	73.12%
		21–23	118	26.88%
	Major	Science	261	59.45%
		Humanities	178	40.55%
	Hometown	Rural	314	71.53%
		Urban	125	28.47%
Study 2	Gender	Male	146	43.20%
		Female	192	56.80%
	Age	18–20	271	80.18%
		21–23	67	19.82%
	Major	Science	211	62.43%
		Humanities	127	37.57%
	Hometown	Rural	264	78.11%
		Urban	74	21.89%

### Research instruments

#### Weight self-stigma scale

The Weight Self-Stigma Questionnaire (WSSQ) developed by Lillis et al. (2010) was used^14^. This questionnaire consists of two factors: self-devaluation and fear of enacted stigma, with a total of 12 items. A Likert 5-point scale was employed, where 1 to 5 represent “strongly disagree” to “strongly agree.” Higher total scores indicate higher levels of weight self-stigma among participants.

#### Physical activity rating scale

The Physical Activity Rating Scale (PARS-3), revised by Liang Deqing (1994), was used to assess participants’ physical exercise levels^21^. This scale evaluates exercise volume based on three dimensions: intensity, frequency, and duration of physical activity, using a Likert 5-point scale. The exercise volume was calculated using the formula: “Exercise Volume = Exercise Frequency × (Exercise Duration − 1) × Exercise Intensity” (with a maximum score of 100 and a minimum score of 0). In this study, the K-S non-parametric test showed significant results (*P* < 0.05, df = 951), and the overall Cronbach’s α coefficient was 0.85.

#### Depression, anxiety, and stress scale

The Depression, Anxiety, and Stress Scale (hereinafter referred to as DASS) developed by Lovibond (1995) was used, consisting of 21 items to assess three types of psychological distress: depression (7 items), anxiety (7 items), and stress (7 items)^22^. A Likert 4-point scale was employed, where 1 to 4 represent “never” to “sometimes.” The score for each subscale was obtained by summing the scores of the corresponding items, with higher scores indicating more severe levels of the respective emotional state.

#### Life satisfaction scale

The Satisfaction with Life Scale (SWLS), developed by Diener et al. (1985), was used, consisting of 5 items^23^. A 7-point scale was employed, where 1 represents “strongly disagree” and 7 represents “strongly agree.” The total life satisfaction score was obtained by summing the scores of all items, with higher scores indicating greater life satisfaction. The Cronbach’s α coefficient for this scale in the Chinese population was 0.78.

### Process of Chinese adaptation for the weight self-stigma scale

Contact was made with the original author via email to obtain authorization for the Chinese adaptation of the original scale, and the Brislin translation model was employed for this adaptation^48^.

1. Translation.

Two experts with both a background in sports psychology and cross-cultural translation experience independently translated the scale into Chinese versions A1 and A2. A third sports psychology expert with overseas study experience compared and integrated A1 and A2 to form the translated version A.

2. Back-translation.

A medical English teacher proficient in medical English and an English postgraduate (who had not been exposed to the original scale) independently back-translated version A into English versions B1 and B2. Through joint discussion by the research team, B1 and B2 were synthesised into the back-translated version B.

3. Integration.

Version B was emailed to the original author for review. Based on the author’s feedback, the research team and a medical English teacher compared and discussed version B with the original scale, finally forming the comprehensive version C.

4. Cultural adaptation.

Six experts were invited to conduct two rounds of expert reviews on version C, including 3 sports psychology experts, 1 health policy expert, and 1 medical expert (all holding master’s degrees or above, with 5 associate senior titles and 1 senior titles, and over 10 years of working experience). The experts evaluated each scale item for semantic equivalence, cultural appropriateness, and clarity of expression based on their professional knowledge and experience, proposing corresponding revisions to form the Chinese version D, which was used for the pre-survey.

### Statistical analysis

#### Reliability and validity testing in study 1

Item Analysis: Item analysis was conducted on the data used for exploratory factor analysis (EFA). (1) Critical Ratio Method: The discriminative power of scale items was tested using the critical ratio (CR) method. The total scores of the scale were ranked from high to low, with the top 27% and bottom 27% classified as the high-score and low-score groups, respectively. An independent samples t-test was performed on the scores of each item based on these groups. Items with a CR > 3 and *P* < 0.05 were considered to have good discriminative power and were retained. (2) Pearson Correlation Coefficient Method: The correlation between each item and the total scale score was assessed using Pearson correlation coefficients. Higher correlations indicated better homogeneity. Items with statistically significant differences (*P* < 0.05) or a correlation coefficient ≥ 0.4 with the total score were retained. Validity Testing: The validity of the WSSQ was evaluated using structural validity, convergent validity, discriminant validity, and criterion validity. Reliability Testing: Reliability was assessed using Cronbach’s α coefficient.

#### Chain mediation analysis in study 2

Common Method Bias Analysis: Harman’s single-factor test was used to assess common method bias. Correlation Analysis: Pearson correlation analysis was conducted to explore the relationships between weight self-stigma and physical exercise, depression-anxiety-stress, and life satisfaction. Chain Mediation Effect Analysis: AMOS 24.0 was used to perform chain mediation effect analysis.

## Results analysis

### Item analysis

The primary objective of item analysis is to assess the suitability and reliability of individual scale items. This process differs from reliability testing, which evaluates the stability of the entire instrument or its composite dimensions. To ensure objectivity, this study employed the extreme group comparison, item-total correlation, and homogeneity tests for statistical analysis. As detailed in Table 2, multiple indicators supported the retention of all items. The Chinese version of the WSSQ demonstrated strong discriminant validity, with critical ratio (CR) values for each item ranging from 7.808 to 21.023 (all *p* < 0.01). Pearson correlation analysis showed that the pre-correction coefficients between individual items and the total score ranged from 0.573 to 0.794 (all *p* < 0.01); post-correction, these values spanned 0.444–0.772, all exceeding the 0.4 threshold. In the homogeneity test, Cronbach’s alpha values upon item deletion ranged from 0.883 to 0.900 (below the 0.900 cut-off), communality values varied from 0.428 to 0.805 (exceeding 0.2), and factor loadings ranged from 0.624 to 0.863 (above 0.45). Collectively, these results justified retaining all 12 items in the scale.

**Table 2:** Summary of item analysis for the WSSQ. Source(s): author’s own creation

Item	Extreme group comparison	Item-total correlation		Homogeneity test			Number of criteria not met	Decision
	Critical ratio (CR)	Before correction	After correction	Cronbach’s alpha if item deleted	Communality	Factor loading		
	≥ 3.000	≥ 0.400	≥ 0.400	≤ 0.900	≥ 0.200	≥ 0.450		
1	16.214***	0.687***	0.686	0.888	0.686	0.766	0	Retain
2	10.564***	0.590***	0.462	0.900	0.428	0.641	0	Retain
3	15.184***	0.686***	0.635	0.891	0.677	0.791	0	Retain
4	7.808***	0.573***	0.444	0.900	0.513	0.715	0	Retain
5	21.023***	0.794***	0.772	0.883	0.798	0.814	0	Retain
6	13.379***	0.732***	0.647	0.890	0.620	0.727	0	Retain
7	17.691***	0.784***	0.734	0.885	0.805	0.863	0	Retain
8	11.861***	0.708***	0.614	0.892	0.707	0.832	0	Retain
9	12.658***	0.705***	0.627	0.891	0.566	0.701	0	Retain
10	13.583***	0.710***	0.668	0.889	0.653	0.760	0	Retain
11	9.317***	0.624***	0.522	0.896	0.431	0.624	0	Retain
12	13.831***	0.678***	0.616	0.892	0.681	0.812	0	Retain

Note: *** *p* < 0.001; ** *p* < 0.01; * *p* < 0.05; # indicates criteria not met. The scale’s alpha value in this table is 0.900.

### Structural validity

#### Exploratory factor analysis

In the initial stage of translating the scale into Chinese, an exploratory analysis without prior assumptions was undertaken to reveal the internal relationships among the items and determine the potential dimensional structure of weight self-stigma among Chinese college students. The primary objective is to reduce the dimensionality of the items and form a set of variables with strong logical connections, ensuring that the structural validity of the scale aligns with the psychological characteristics of the target group (such as Chinese college students).

1. Factor analysis feasibility assessment.

Firstly, the feasibility of factor analysis was assessed using the Kaiser-Meyer-Olkin (KMO) test and Bartlett’s test of sphericity. According to conventional standards in social science research^24^a KMO value of ≥ 0.6 indicates that the data is suitable for factor analysis, with an ideal value exceeding 0.8 (the higher the value, the greater the common variance among the items). A Bartlett’s test with a P-value < 0.001 rejects the null hypothesis of “no correlation among variables.” In this study, the KMO sampling adequacy measure was 0.904 (significantly higher than 0.8), and the P-value for Bartlett’s test of sphericity was < 0.001, indicating a strong correlation among the items and fulfilling the prerequisites for exploratory factor analysis (EFA).

2. Factor extraction technique and standards.

**Fig. 1:**
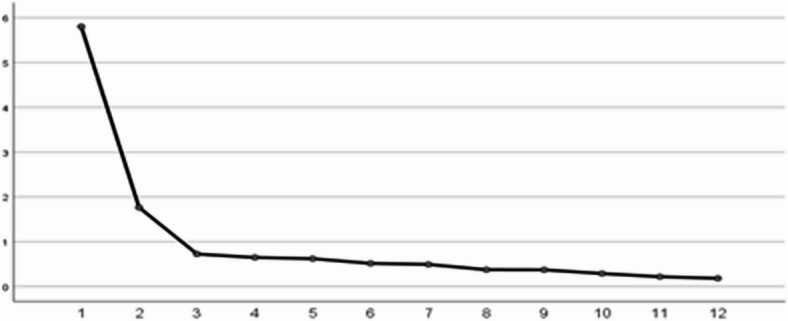
Scree plot of exploratory factor analysis for the WSSQ scale. Source(s): Author’s own creation

Principal Component Analysis (PCA) was employed as the data reduction technique, as it is well-suited for exploratory research scenarios. By maximizing the total variance of the original data (including both common and unique variances), PCA unbiasedly reveals the underlying factor structure^20^. Factor extraction followed the Kaiser criterion (eigenvalue ≥ 1). This criterion ensures that each extracted factor explains no less variance than the average variance of a single variable (since the variance of a standardized variable is 1), thereby preventing the inclusion of spurious factors that merely reflect random noise^24^. Meanwhile, a scree plot was used for supplementary validation: with the factor number on the horizontal axis and the variance explained rate on the vertical axis, Fig. 1 shows that the curve flattens after extracting the first two factors (forming an “elbow inflection point”), which is consistent with the result of eigenvalues ≥ 1, further supporting the rationality of the number of factors and avoiding potential biases from a single numerical criterion.

3. Factor rotation and structure clarification.

To enhance factor interpretability, the Varimax Rotation method was adopted. This method assumes independence among factors. By adjusting the distribution of factor loadings, it aims to make each item have a high loading (> 0.5) on as few factors as possible and a loading approaching 0 on other factors, thereby forming a “simple structure“^20^. After rotation, two common factors were extracted: Factor 1 (with an eigenvalue of 5.801 and a variance contribution rate of 48.34%) includes items such as “I always return to an overweight state.” It focuses on individuals’ internal negation of their own body weight and is named “Self-deprecation.” Factor 2 (with an eigenvalue of 1.765 and a variance contribution rate of 14.71%) includes items such as “People discriminate against me because of my weight problem.” It emphasizes concerns about negative external evaluations and is named “Fear of Enacted Stigma.” The cumulative variance contribution rate is 63.05%, indicating that these two factors capture over 60% of the common variance in the data and possess strong explanatory power.

4. Item validity and structural reliability.

As presented in Table 3, all items’ standardized factor loadings (0.624 - 0.863) exceeded the 0.5 threshold. Following preliminary item screening, EFA identified no items for deletion, reinforcing the alignment between items and the factor structure. This provided a robust initial model for subsequent confirmatory factor analysis (CFA).

**Table 3:** Exploratory factor analysis of the WSSQ scale. Source(s): author’s own creation

Item	Factor 1	Factor 2
1. I’ll always go back to being overweight		0.766
2. I caused my weight problems		0.641
3. I feel guilty because of my weight problems		0.791
4. I became overweight because I’m a weak person		0.715
5. I would never have any problems with weight if I were stronger		0.814
6. I don’t have enough self-control to maintain a healthy weight		0.727
7. I feel insecure about others’ opinions of me	0.863	
8. People discriminate against me because I’ve had weight problems	0.832	
9. It’s difficult for people who haven’t had weight problems to relate to me	0.701	
10. Others will think I lack self-control because of my weight problems	0.760	
11. People think that I am to blame for my weight problems	0.624	
12. Others are ashamed to be around me because of my weight	0.812	
Eigenvalue	5.801	1.765
% of variance	48.341	14.708
Cumulative %	48.341	63.050

#### Confirmatory factor analysis

**Fig. 2:**
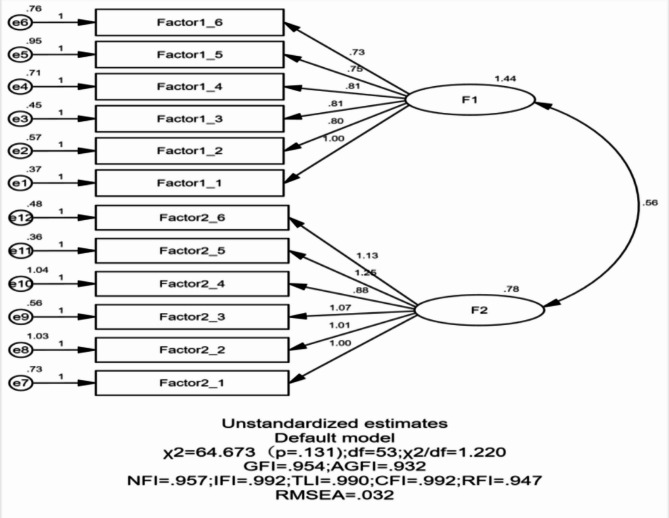
Confirmatory factor analysis of the WSSQ Scale. Source(s): Author’s own creation

Confirmatory factor analysis (CFA) was conducted on the two-factor structure derived from exploratory factor analysis (EFA), aiming to verify model-data fit and cross-sample stability of the scale’s dimensional structure. As shown in Fig. 2, the initial model evaluation revealed factor loadings of 0.60–0.89 for all items, with all parameters statistically significant (*P* < 0.05), meeting preliminary evaluation criteria.

Using the EFA-derived two-factor first-order model, parameter estimation was performed via maximum likelihood estimation^47^. Table 4 shows that the model fit indices met acceptable thresholds: χ^2^/df < 3, RMSEA < 0.08, SRMR < 0.08, and NFI, IFI, TLI, CFI, RFI, GFI > 0.9^48^. The hypothesized CFA model demonstrated good fit to the observed data, necessitating further examination of its convergent and discriminant validity.

**Table 4:** CFA fit indices for the WSSQ. Source(s): author’s own creation

Index	Benchmark	Actual measured results
X^2^/df	< 2.00 Excellent; < 3.00 Acceptable	1.22
RMSEA	< 0.08 Acceptable; <0.05 Excellent	0.032
SRMR	< 0.08 Acceptable; <0.05 Excellent	0.036
NFI	> 0.90 Acceptable; ≥0.95 Excellent	0.957
IFI	> 0.90 Acceptable; ≥0.95 Excellent	0.992
TLI	> 0.90 Acceptable; ≥0.95 Excellent	0.990
RFI	> 0.90 Acceptable; ≥0.95 Excellent	0.947
CFI	> 0.90 Acceptable; ≥0.95 Excellent	0.992
GFI	> 0.90 Acceptable; ≥0.95 Excellent	0.954

### Convergent validity

Convergent validity quantifies the correlation among indicator variables measuring the same latent construct. Higher inter-variable correlations lead to greater factor loadings, indicating stronger convergent validity. This construct validity is typically evaluated using two key metrics: Average Variance Extracted (AVE) and Composite Reliability (CR). An AVE value exceeding 0.50 and a CR value above 0.70 are considered satisfactory^20^. Table 5 presents the convergent validity indices for the Self-Depreciation and Fear of Enacted Stigma. The AVE values of 0.562 and 0.605, respectively, surpass the 0.5 threshold, confirming robust convergent validity. Additionally, the CR values of 0.884 and 0.901 for these dimensions reflect excellent composite reliability. Collectively, these results demonstrate that the scale exhibits good convergent validity.

**Table 5:** Convergent validity of the WSSQ scale. Source(s): author’s own creation

Latent Variable	Item	Standardized factor loading	CR	AVE
Self-Deprecation	I’ll always go back to being overweight	0.718	0.884	0.562
	I caused my weight problems	0.66		
	I feel guilty because of my weight problems	0.783		
	I became overweight because I’m a weak person	0.603		
	I would never have any problems with weight if I were stronger	0.877		
	I don’t have enough self-control to maintain a healthy weight	0.823		
Fear of Enacted Stigma	I feel insecure about others’ opinions of me	0.891	0.901	0.605
	People discriminate against me because I’ve had weight problems	0.787		
	It’s difficult for people who haven’t had weight problems to relate to me	0.821		
	Others will think I lack self-control because of my weight problems	0.757		
	People think that I am to blame for my weight problems	0.68		
	Others are ashamed to be around me because of my weight	0.712		

### Discriminant validity

Discriminant validity refers to the degree of dissimilarity (i.e., low correlation) between latent traits represented by different constructs, ensuring each construct is distinct from others. The most common approach tests whether the square root of each construct’s Average Variance Extracted (AVE) exceeds its inter-construct correlation coefficient (equivalently, AVE > correlation²), reflecting the theoretical principle that a construct should explain more variance in its items than other constructs do^20^. Table 6 presents discriminant validity results for the Self-Depreciation and Fear of Enacted Stigma constructs. The AVE square roots were 0.750 (Self-Depreciation) and 0.778 (Fear of Enacted Stigma), both exceeding the inter-construct correlation (0.53). This confirms good discriminant validity between the two dimensions.

**Table 6:** Discriminant validity of the WSSQ scale. Source(s): author’s own creation

Latent variable	Self-deprecation	Fear of enacted stigma
Self-Deprecation	**0.750**	
Fear of Enacted Stigma	0.528	**0.778**

Significant values are in [bold].

Note: The diagonal values represent the square roots of the AVE for each latent variable.

### Criterion validity

Criterion validity is assessed by correlating the new scale with a recognized valid criterion scale, with strength measured by their score correlation coefficient^20^. A higher coefficient indicates better criterion validity. In this study, the WSSQ was correlated with the DASS. Table 7 shows significant positive correlations between the WSSQ’s two factors, total scale, and the DASS. Additionally, BMI correlated significantly with the Self-Depreciation factor. Notably, this contrasts with prior literature: no significant correlations emerged between BMI and the Fear of Enacted Stigma subscale or total WSSQ.

**Table 7:** Criterion validity of the WSSQ scale. Source(s): author’s own creation

	Variables	M	SD	1	2	3	4	5	6	7	8
1	WSSQ—Total	2.55	0.87	-							
2	WSSQ—Self-D	2.54	0.99	0.850**	-						
3	WSSQ—FES	2.56	1.03	0.862**	0.466**	-					
4	Depression Anxiety Stress	1.81	0.66	0.644**	0.568**	0.536**	-				
5	Depression	1.81	0.78	0.597**	0.502**	0.519**	0.836**	-			
6	Anxiety	1.85	0.82	0.516**	0.465**	0.419**	0.835**	0.573**	-		
7	Stress	1.77	0.81	0.489**	0.444**	0.395**	0.811**	0.516**	0.492**	-	
8	BMI	26.84	2.27	0.126	0.141*	0.076	0.007	-0.022	0.064	-0.026	-

Note: *** *p* < 0.001; ** *p* < 0.01; * *p* < 0.05.

### Internal consistency reliability

As shown in Table 8, internal consistency reliability, also known as homogeneity reliability, refers to the degree of consistency among all items within a scale. The Cronbach’s alpha coefficient ranges between 0 and 1, with α ≥ 0.9 indicating excellent reliability of the scale^21^. In this study, the internal consistency of the Weight Self-Stigma Questionnaire (WSSQ) was examined, and the results showed that the overall Cronbach’s alpha coefficient for the WSSQ scale was 0.899. Specifically, the Cronbach’s alpha coefficients for the Self-Deprecation and the Fear of Enacted Stigma were 0.875 and 0.892 respectively.

**Table 8:** Reliability analysis of the WSSQ scale. Source(s): author’s own creation

Scale	Dimension	Dimension Cronbach’s α	Scale Cronbach’s α
WSSQ	Self-Deprecation	0.875	0.899
	Fear of Enacted Stigma	0.892	

### Study 2 analysis

#### Common method bias control and testing

##### Common method bias

To address potential common method bias, this study employed procedural controls and Harman’s single-factor test. Procedural controls included informing participants of survey anonymity and confidentiality prior to administration. For the Harman test, all items from the four scales were subjected to exploratory factor analysis. Results showed that the unrotated solution yielded seven factors with eigenvalues > 1, explaining 67.352% of total variance. The first factor accounted for 34.812% of variance—below the 40% threshold—indicating no severe common method bias.

#### Descriptive statistics and correlation analysis of research variables

Table 9 presents means, standard deviations, and Pearson correlation coefficients among weight self-stigma, physical exercise, life satisfaction, and depression-anxiety-stress total score. Correlation analysis revealed significant associations: Weight self-stigma was negatively correlated with life satisfaction (*r* = − 0.538, *p* < 0.01) and physical exercise (*r* = − 0.452, *p* < 0.01), but positively correlated with depression-anxiety-stress (*r* = 0.480, *p* < 0.01). Physical exercise was positively correlated with life satisfaction (*r* = 0.599, *p* < 0.01) and negatively correlated with depression-anxiety-stress (*r* = − 0.584, *p* < 0.01). Life satisfaction was negatively correlated with depression-anxiety-stress (*r* = − 0.598, *p* < 0.01). All variables exhibited significant correlations, providing a preliminary foundation for subsequent mediation effect analysis.

**Table 9:** Pearson correlation coefficients among weight self-stigma, physical exercise, depression-anxiety-stress, and life satisfaction (*N* = 338). Source(s): author’s own creation

		M	SD	1	2	3	4
1	Weight self- stigmatization	2.67	0.83	-			
2	Physical exercise	2.59	1.83	− 0.452^**^	-		
3	Life satisfaction	3.93	1.50	− 0.538^**^	0.599^**^	-	
4	Depression Anxiety Stress	1.63	0.58	0.480^**^	− 0.584^**^	− 0.598^**^	-

Note: *** *p* < 0.001; ** *p* < 0.01; * *p* < 0.05.

### The relationship between weight self-stigma and life satisfaction: testing the chain mediation effect

#### Model fit analysis

**Fig. 3:**
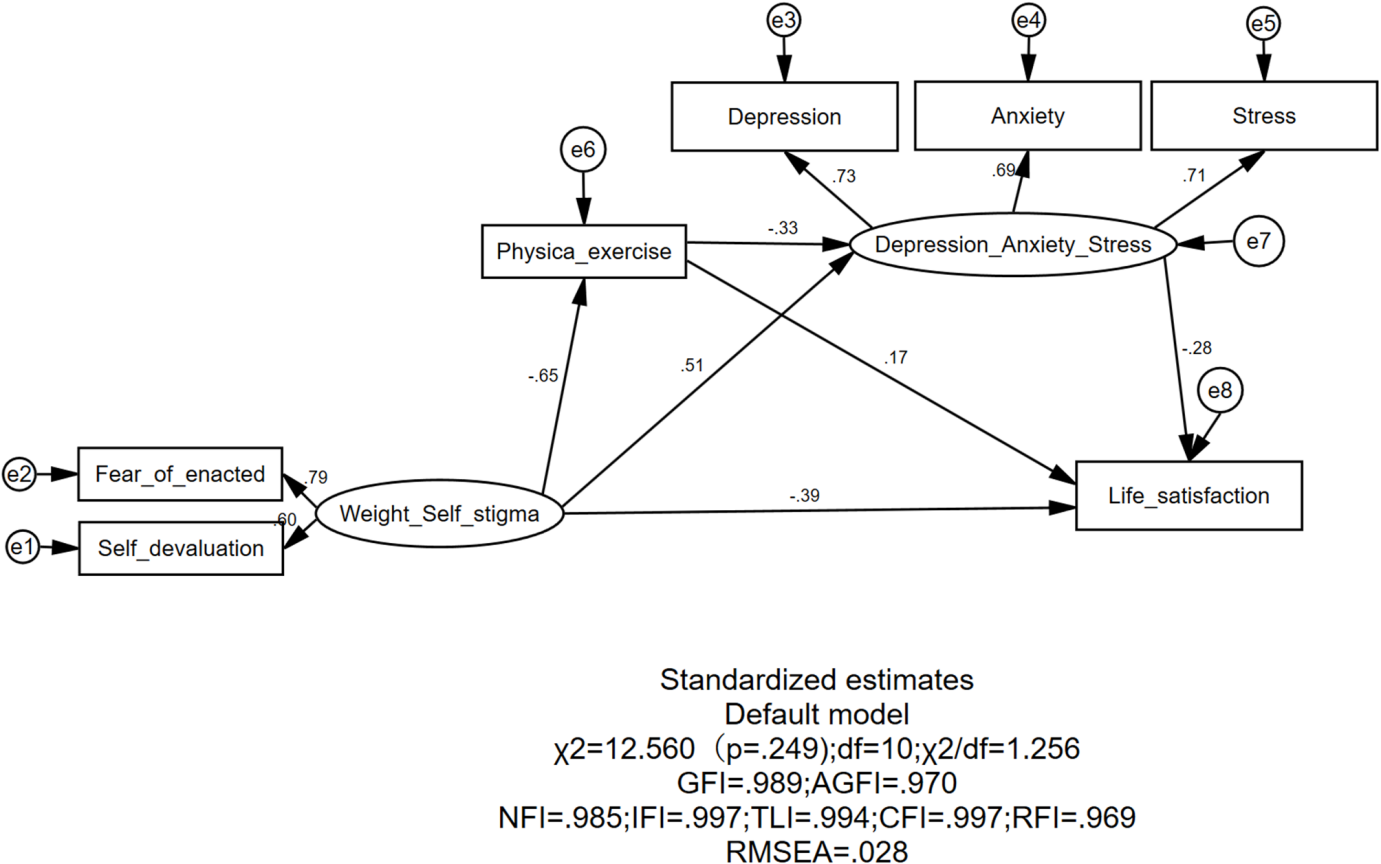
Model diagram of the chain mediation effect of physical exercise and depression-anxiety-stress in the relationship between weight self-stigma and life satisfaction. Source(s): Author’s own creation

Weight self-stigma was treated as the independent variable and life satisfaction as the dependent variable to test whether physical exercise and depression-anxiety-stress chain-mediate the effect of weight self-stigma on college students’ life satisfaction. The measurement model was analyzed to validate the structural model. As shown in Fig. 3; Table 10, standardized loadings of observed variables on latent variables and model fit indices are presented. Fit indices included: χ^2^/df = 1.26, RMSEA = 0.028 (< 0.08), SRMR = 0.021 (< 0.05), NFI = 0.985, IFI = 0.997, RFI = 0.969, CFI = 0.997, TLI = 0.994, and GFI = 0.989. All exceeded the 0.90 threshold^47^indicating excellent model fit.

**Table 10:** Fit indices for the chain mediating effect of physical exercise and depression-anxiety-stress in the relationship between weight self-stigma and life satisfaction. Source(s): author’s own creation

Index	Benchmark	Actual measured results
X^2^/df	< 2.00 Excellent; < 3.00 Acceptable	1.256
RMSEA	< 0.08 Acceptable; <0.05 Excellent	0.028
SRMR	< 0.08 Acceptable; <0.05 Excellent	0.021
NFI	> 0.90 Acceptable; ≥0.95 Excellent	0.985
IFI	> 0.90 Acceptable; ≥0.95 Excellent	0.997
TLI	> 0.90 Acceptable; ≥0.95 Excellent	0.994
CFI	> 0.90 Acceptable; ≥0.95 Excellent	0.997
RFI	> 0.90 Acceptable; ≥0.95 Excellent	0.969
GFI	> 0.90 Acceptable; ≥0.95 Excellent	0.989

#### Direct effect test

As shown by the path coefficients in Table 11, weight self-stigma had significant negative and positive effects on physical exercise (β = -0.647, *p* < 0.001) and depression, anxiety, and stress respectively, and had a significant negative direct effect on life satisfaction (β = -0.390, *p* < 0.001). That is, college students with higher levels of self-stigma had lower frequencies of physical exercise, higher levels of depression, anxiety, and stress, and lower life satisfaction, supporting Hypothesis 2. Path analysis of physical exercise and depression, anxiety, and stress showed that physical exercise significantly reduced depression, anxiety, and stress (β = -0.332, *p* < 0.001) and significantly improved life satisfaction (β = 0.170, *p* < 0.01); depression, anxiety, and stress significantly reduced life satisfaction (β = -0.277, *p* < 0.01). These results indicate that higher frequencies of physical exercise are associated with lower levels of negative emotions and higher life satisfaction among college students; conversely, higher levels of depression, anxiety, and stress are associated with lower life satisfaction, which is consistent with theoretical expectations.

**Table 11:** Path coefficients of the chain mediation model of physical exercise and depression-anxiety-stress between weight self-stigma and life satisfaction.

Path	β	S.E.	C.*R*.	*P*
Weight Self-Stigma → Physical Exercise	-0.647	0.155	-8.141	0.000
Weight Self-Stigma → Depression, Anxiety, Stress	0.513	0.097	4.752	0.000
Weight Self-Stigma → Life Satisfaction	-0.390	0.290	-3.636	0.000
Physical Exercise → Depression, Anxiety, Stress	-0.332	0.039	-3.877	0.000
Physical Exercise → Life Satisfaction	0.170	0.090	2.625	0.009
Depression, Anxiety, Stress → Life Satisfaction	-0.277	0.291	-2.871	0.004

Note: *** *p* < 0.001; ** *p* < 0.01; * *p* < 0.05 Source(s): Author’s own creation.

#### Mediation effect testing using the bootstrap method

A bias-corrected nonparametric percentile Bootstrap approach was employed for mediational effect testing. With a specified Bootstrap resampling size of 5,000, 95% confidence intervals (CIs) were calculated, where CIs excluding zero indicate statistical significance. Compared to traditional parametric tests (e.g., Sobel test), the Bootstrap method constructs sampling distributions through repeated resampling, eliminating the need for normality assumptions about variables. This makes it particularly suitable for small samples or asymmetrically distributed data, enabling more robust estimation of confidence intervals for indirect effects^47^. Given the multiple mediational pathways and potential complex distributional characteristics of variables in this study, using the Bootstrap method enhanced test power and reduced the risk of Type I errors, as shown in Table 12. The results of the mediational effect test revealed three significant indirect effects through which weight self-stigma influenced college students’ life satisfaction: The indirect effect of the pathway “weight self-stigma → physical exercise → life satisfaction” was − 0.110 (SE = 0.046), with a 95% CI of [-0.202, -0.021], supporting Hypothesis 3. The path coefficient indicated that each unit increase in weight self-stigma indirectly reduced life satisfaction by 0.110 units through decreased physical exercise (β = -0.110, *p* < 0.05). This finding confirms the theoretical mechanism that weight self-stigma undermines subjective well-being by suppressing healthy behaviors. The indirect effect of the pathway “weight self-stigma → depression, anxiety, and stress → life satisfaction” was − 0.142 (SE = 0.058), with a 95% CI of [-0.285, -0.051], supporting Hypothesis 4. The path coefficient showed that each unit increase in weight self-stigma indirectly reduced life satisfaction by 0.142 units through intensified negative emotions (β=-0.142, *p* < 0.01). This pathway had the largest effect size, highlighting the core role of psychological-emotional mechanisms. The chained mediational effect of the pathway “weight self-stigma → physical exercise → depression, anxiety, and stress → life satisfaction” was − 0.059 (SE = 0.017), with a 95% CI of [-0.133, -0.011], supporting Hypothesis 5. The path coefficient demonstrated that weight self-stigma ultimately reduced life satisfaction by 0.059 units through the chained transmission of “decreased exercise → heightened negative emotions” (β=-0.059, *p* < 0.05).

**Table 12:** Bootstrap mediation effect Estimation for the chain mediation model of physical exercise and depression-anxiety-stress between weight self-stigma and life satisfaction. Source(s): author’s own creation

95%CI							
Type	Mediation path	Standardized effect size	Effect proportion	SE	Lower bound	Upper bound	P
Direct effect	Weight Self- Stigma→Life Satisfaction	-0.390	55.63%	0.115	-0.629	-0.173	0.001
Mediation effects	Weight Self- Stigma→Physical Exercise→Life Satisfaction	-0.110	15.69%	0.046	-0.202	-0.021	0.023
	Weight Self- Stigma→Depression, Anxiety, Stress→Life Satisfaction	-0.142	20.26%	0.058	-0.285	-0.051	0.009
	Weight Self- Stigma→Physical Exercise→Depression, Anxiety, Stress→Life Satisfaction	-0.059	8.42%	0.017	-0.133	-0.011	0.011
Total mediation effect		-0.311	44.37%	0.067	-0.489	-0.153	0.004
Total Effect		-0.701	100%	0.071	-0.784	-0.609	0.001

Note: *** *p* < 0.001; ** *p* < 0.01; * *p* < 0.05.

## Discussion

### Study 1

In Study 1, the psychometric properties of the Chinese-translated WSSQ. Specifically, the Weight Self-Stigma Scale was divided into two dimensions: Self-Depreciation and Fear of Enacted Stigma, forming a 12-item Weight Self-Stigma Scale. Based on item analysis results, an exploratory factor analysis (EFA) was conducted on the 12 items. Indicators including the critical ratio method, corrected item-total correlations, Cronbach’s α values after item deletion, communality, and factor loadings suggested no need for further item deletion, retaining all 12 items. The EFA showed a KMO value of 0.904 and a Bartlett’s test statistic significant at *p* < 0.001, meeting standard criteria. Using principal component analysis with a criterion of eigenvalue ≥ 1, the EFA preliminarily confirmed a two-dimensional structure for the scale.

Confirmatory factor analysis (CFA) of the two-dimensional model further demonstrated that the Chinese version of the WSSQ exhibited satisfactory validity and reliability among university students. All fit indices met psychometric standards: χ^2^/df = 1.22, RMSEA = 0.032, SRMR = 0.036, NFI = 0.957, IFI = 0.992, TLI = 0.990, CFI = 0.992, and GFI = 0.954. For convergent validity, the average variance extracted (AVE) values were 0.562 and 0.605 (both > 0.5), and composite reliability (CR) values were 0.884 and 0.901 (both > 0.7). For discriminant validity, the square roots of the AVEs were 0.750 (Self-Depreciation) and 0.778 (Fear of Enacted Stigma), both higher than the inter-factor correlation coefficient of 0.53. These results supported the two-factor structure of the Chinese WSSQ. The internal consistency (Cronbach’s α = 0.899) was consistent with the original English version^15^ and German, French, Indonesian, and Italian versions, all of which reported Cronbach’s α > 0.8^26–29^, Supporting Hypothesis 1.

Additionally, criterion validity analysis showed a significant positive correlation between the WSSQ and DASS-21 (*r* = 0.644, *p* < 0.01), consistent with findings in the Indonesian version (*r* = 0.280, *p* < 0.001)^27^. The non-significant correlation between the WSSQ and BMI (*r* = 0.126, *p* > 0.05) aligned with Italian research (*r* = 0.081, *p* > 0.05)^29^ but contrasted with Indonesian (*r* = 0.390, *p* < 0.001) and English (*r* = 0.40, *p* < 0.001) results^15,27^. The non-significant correlation between the WSSQ-FES subscale and BMI (*r* = 0.076, *p* > 0.05) matched the Italian version (*r* = 0.134, *p* > 0.05)^29^ but differed from Indonesian (*r* = 0.170, *p* < 0.001) and English (*r* = 0.40, *p* < 0.001) findings^15,27^. Notably, the WSSQ-Self-D subscale showed a significant correlation with BMI (*r* = 0.141, *p* < 0.05), consistent with Indonesian (*r* = 0.500, *p* < 0.001) and English (*r* = 0.300, *p* < 0.001) results^15,27^. These findings suggest potential cross-cultural and cross-population influences on the relationship between BMI and weight self-stigma.

### Study 2

In Study 2, the relationship between weight-based self-stigma, physical exercise, depression, and quality of life was investigated, with a focus on the mechanisms underlying the association between weight-based self-stigma and quality of life among Chinese college students. By introducing physical exercise and depression as mediating variables, a chain mediation model was constructed and validated to explore the direct and indirect pathways through which weight-based self-stigma influences the quality of life of Chinese college students. The total effect of weight-based self-stigma on the quality of life of Chinese college students was − 0.701, with a direct effect of -0.390 and a total indirect (mediation) effect of -0.311. Therefore, the results indicate that weight-based self-stigma significantly and negatively predicts the quality of life of Chinese college students. Specifically: Physical exercise mediates the relationship between weight-based self-stigma and quality of life. Depression, anxiety, and stress mediate the relationship between weight-based self-stigma and quality of life. Physical exercise and depression, anxiety, and stress together play a chain-mediating role in the relationship between weight-based self-stigma and quality of life. These findings highlight the complex mechanisms through which weight-based self-stigma impacts the quality of life of Chinese college students, emphasizing the roles of both behavioral (physical exercise) and psychological (depression, anxiety, and stress) factors as mediators.

### The impact of weight-based self-stigma on college students’ quality of life satisfaction

The study found that as the level of weight-based self-stigma among college students increases, their quality of life significantly decreases. This indicates that weight-based self-stigma can significantly and negatively predict the quality of life of college students, which aligns with the findings of Khodari et al. (2017). It confirms that college students with higher weight-based self-stigma scores tend to have lower quality of life satisfaction, demonstrating a significant negative correlation between weight-based self-stigma and quality of life satisfaction^29^. Weight-based self-stigma makes college students highly susceptible to negative emotions. Overweight or obese students, influenced by society’s preference for slim body types, internalize negative societal evaluations of obesity, often falling into feelings of inferiority, anxiety, and depression^30^. These persistent emotional struggles severely affect their daily mood and emotional stability, leading to excessive sensitivity and nervousness in social situations, which hinders normal interpersonal interactions^31^. Additionally, weight-based self-stigma is closely linked to self-identity and self-esteem, causing students to feel dissatisfied with their self-image and experience reduced self-esteem. When facing academic or life challenges, they may lack confidence due to self-devaluation, avoiding opportunities for growth such as participating in competitions or running for positions in student organizations^32,33^. Moreover, due to weight-based self-stigma, some students actively avoid social activities, fearing judgment, ridicule, or discrimination based on their body size. This leads to reduced participation in gatherings and group activities^34^. Over time, their social circles shrink, and the development of interpersonal relationships is hindered. For example, frequent absences from dormitory activities may result in estranged relationships with roommates. Even when participating in social events, their excessive concern about others’ perceptions of their weight makes them appear reserved, preventing them from expressing their true selves and building deep, meaningful relationships^35^. Furthermore, some students cope with the psychological pressure of weight-based self-stigma through unhealthy eating behaviors^36^. Some may overeat to alleviate emotional distress, consuming large amounts of high-calorie foods in a short period, which exacerbates weight issues^37,38^. Others may resort to extreme dieting in an attempt to lose weight quickly, damaging their physical health and potentially leading to eating disorders such as anorexia nervosa or bulimia^39^. Additionally, weight-based self-stigma causes some students to avoid physical activity due to fear of being judged or mocked in sports settings^40^. A lack of exercise not only hinders weight control but also negatively impacts physical fitness and overall health. Over time, declining physical function makes them more prone to illness, further disrupting their academic and daily lives. Therefore, weight-based self-stigma among college students can positively predict their quality of life satisfaction, highlighting the profound and multifaceted impact of this stigma on their well-being.

### The chained mediating role of physical exercise on depression, anxiety, and stress

Weight self-stigma can lead college students to reduce physical exercise, thereby affecting mental health (e.g., depression, anxiety, and stress) and ultimately diminishing quality of life. Weight self-stigma may cause individuals to develop avoidance behaviors toward physical activity. This avoidance is not entirely due to an aversion to exercise itself but rather stems from anticipated negative evaluations from others or dissatisfaction with one’s body image in exercise settings. Such behavior not only deprives individuals of the physical benefits of exercise but may also exacerbate weight-related issues, creating a vicious cycle. Physical exercise serves as a critical protective factor for mental health^41^. Regular exercise significantly reduces levels of depression, anxiety, and stress. During exercise, the body releases endorphins, which alleviate psychological tension^42^. When college students reduce physical activity due to weight self-stigma, improvements in physical function and metabolic rates are hindered. This not only worsens weight management challenges but also weakens the body’s ability to cope with stress. Psychologically, reduced exercise limits the brain’s production of mood-enhancing neurotransmitters like endorphins and dopamine. Endorphins, often called “feel-good hormones,” and dopamine, linked to pleasure and motivation, are crucial for emotional regulation. Insufficient secretion of these chemicals makes individuals more vulnerable to negative emotions^43^.Furthermore, physical exercise enhances self-esteem and self-efficacy, empowering individuals to navigate life’s challenges^44^. However, when weight self-stigma diminishes exercise engagement, this protective mechanism weakens, leaving mental health more vulnerable. Depression, anxiety, and stress are key factors that compromise quality of life. When college students reduce exercise due to weight self-stigma, they risk entering a downward spiral of mental health. Depression can sap interest in daily life, anxiety fuels excessive worry about the future, and chronic stress erodes coping capacities^45^. These emotional states not only harm mental health but also spill over into academic performance, social relationships, and daily functioning, leading to a marked decline in quality of life. Long-term exposure to depression, anxiety, and stress impairs concentration and academic efficiency, resulting in poorer grades and eroded confidence in learning abilities. Socially, negative emotions may heighten sensitivity and withdrawal, causing missed social opportunities and strained relationships. Both academic setbacks and social isolation further reduce life satisfaction, compounding the decline in overall well-being. Without intervention, this vicious cycle may inflict lasting harm on the physical and mental health of college students.

### Limitations of the study

This study has the following limitations. First, the sample was sourced from universities in a single region, and due to the homogeneity of regional culture and educational backgrounds, the universality of the research findings for college students nationwide may be limited. However, as an exploratory attempt at localized scale validation, this study still provides important initial evidence for subsequent multi-centre research. Second, the study did not conduct stratified analyses of demographic variables such as gender, major, and body mass index (BMI). Future research could further carry out multi-group comparisons to deepen the understanding of the scale’s measurement invariance and the cross-group applicability of the model. Additionally, while the cross-sectional data design revealed statistical associations between variables, inference of causal relationships requires caution. Follow-up research should validate the dynamics of the mechanism through longitudinal tracking studies and randomized controlled trials.

## Conclusions

This study assessed the Weight - Stigma Scale’s reliability and validity through exploratory and confirmatory factor analyses. Results showed it has two dimensions: fear of enacted stigma and self - depreciation. With good fit and internal consistency, it’s suitable for Chinese college students. Colleges can include this scale in routine mental health assessments to spot students with high weight stigma early. Targeted counselling can then be provided to help them address weight - related issues and prevent mental health problems. Further analysis revealed weight stigma directly lowers life satisfaction and indirectly does so through a chain - mediated path of decreased physical exercise leading to increased depression, anxiety, and stress. This highlights the importance of physical exercise for both physical and mental health, and quality of life. Accordingly, colleges should promote physical exercise more and create a positive campus - wide sports culture. They can also offer personalised exercise plans for students with high weight stigma, encouraging them to exercise more to enhance their physical and mental well - being.

## Data Availability

All data generated or analysed during this study are included in this published article.
